# The novel role of MDM2 in the diagnosis and treatment of dedifferentiated liposarcoma

**DOI:** 10.3389/fonc.2024.1466399

**Published:** 2024-10-18

**Authors:** Jiro Ichikawa, Tomonori Kawasaki, Kojiro Onohara, Satoshi Kanno, Masanori Wako, Satoshi Ochiai, Kaoru Aoki, Hirotaka Haro

**Affiliations:** ^1^ Department of Orthopaedic Surgery, Interdisciplinary Graduate School of Medicine, University of Yamanashi, Chuo, Yamanashi, Japan; ^2^ Department of Pathology, Saitama Medical University International Medical Center, Kawagoe, Saitama, Japan; ^3^ Department of Radiology, Interdisciplinary Graduate School of Medicine, University of Yamanashi, Chuo, Yamanashi, Japan; ^4^ Department of Orthopaedic Surgery, National Hospital Organization (NHO) Kofu National Hospital, Kofu, Yamanashi, Japan; ^5^ Physical Therapy Division, School of Health Sciences, Shinshu University, Nagano, Japan

**Keywords:** dedifferentiated liposarcoma, immunohistochemistry, fluorescence *in situ* hybridization, MDM2, extraskeletal osteosarcoma

## 1 Introduction

Dedifferentiated liposarcoma (DDLPS) is a subtype of liposarcoma that frequently occurs in the retroperitoneum. Approximately 10% of atypical lipomatous tumors/well-differentiated liposarcomas (ALTs/WDLPSs) are dedifferentiated, with one risk factor being a retroperitoneal location (1). DDLPS exhibits heterogeneous differentiation, including myogenic or osteosarcomatous/chondrosarcomatous elements (1). The magnetic resonance imaging (MRI) findings of DDLPS show diversity because the degree of fat components within DDLPS may vary in each case, suggesting that the diagnostic power of MRI is limited and that histopathological findings are needed (2). The importance of MDM2 and CDK4 in histopathological diagnosis, especially in immunohistochemistry (IHC) and fluorescence *in situ* hybridization (FISH), remains unclear, and the importance of these two markers as therapeutic targets has been recently highlighted (3, 4). We read, with great interest, the article by Dr. Sosnowska-Sienkiewicz and colleagues titled “A Rare Case of Dedifferentiated Liposarcoma with Osteosarcomatous Differentiation-Diagnostic and Therapeutic Challenges” published in *Diseases* (5). Owing to this well-written paper’s high value in the field, we would like to comment on it from the perspective of our sarcoma team, with recent diagnostic and therapeutic developments to add.

Our paper discusses the following topics: 1) the clinical features of heterogeneous differentiation; 2) the role of MRI in the diagnosis of DDLPS; 3) the role of histopathology in the diagnosis of DDLPS; and 4) treatment strategies.

## 2 Discussion

### 2.1 Clinical features of heterogeneous differentiation

**Figure 1:**
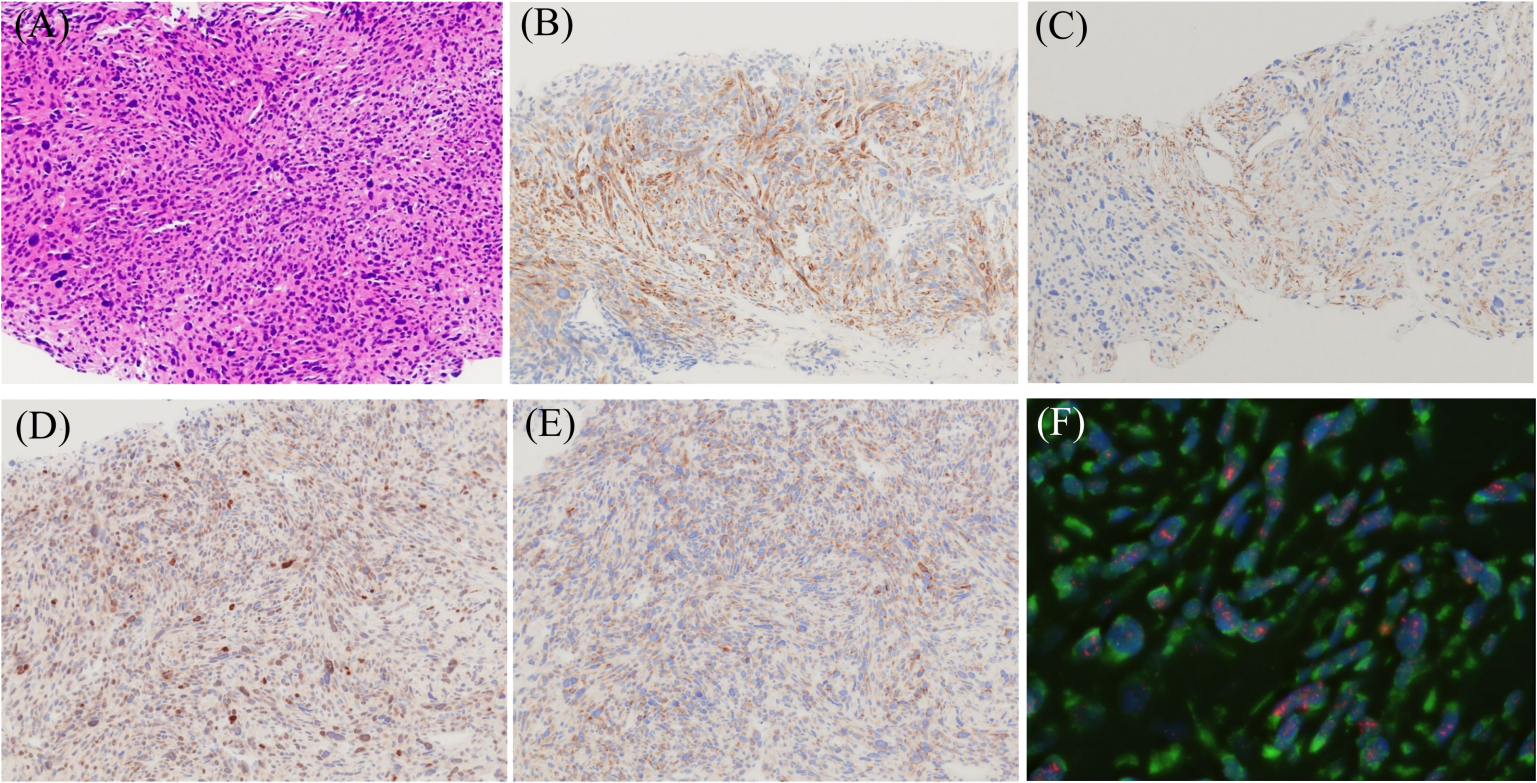
Morphological, immunohistochemical (IHC), and fluorescence *in situ* hybridization (FISH) images of dedifferentiated liposarcoma showing leiomyosarcoma phenotype. **(A)** Hematoxylin and eosin staining image (magnification ×200). Immuno-positivities for h-caldesmon **(B)**, α-SMA **(C)**, MDM2 **(D)**, and CDK4 **(E)** (magnification ×200). FISH analysis demonstrates MDM2 amplification **(F)**. Red: MDM2 locus at 12q15, Green: centromere of chromosome 12 (SE 12/D12Z3). (objective lens magnification ×64).

Approximately 5–10% of patients with DDLPS show heterogeneous differentiation (1). The most common differentiation is myogenic (**Figure 1**); however, osteosarcomatous/chondrosarcomatous and angiosarcomatous elements, although very rare, have also been reported (1, 6). Myogenic differentiation in DDLPS has been investigated. Binh et al. (7) reported that in 27 cases of DDLPS, myogenic differentiation did not affect prognosis or metastasis compared to conventional DDLPS. In contrast, Gronchi et al. (6) reported that in 52 cases of DDLPS, myogenic differentiation in the retroperitoneum, in addition to the Fédération Nationale des Centres de Lutte Contre le Cancer (FNCLCC) grade, affected overall survival (OS) and distant metastasis. The differences between these two reports are as follows: 1) case number and 2) tumor location (Binh’s cases originated in the internal trunk). Additionally, Kurzawa et al. proposed a combined myogenic differentiation score based on IHC staining of smooth muscle actin and desmin, evaluated by scoring intensity and focality. The combined myogenic differentiation score correlated with disease-free survival and OS, suggesting that further research could develop its clinical application (8). In osteogenic differentiation, 36 cases of DDLPS with osteogenic differentiation were reported (9); retroperitoneal location correlated with local recurrence-free survival and distant metastasis-free survival, but not with OS. In contrast, the FNCLCC grade correlated with OS and distant metastasis-free survival, suggesting a trend similar to that of myogenic differentiation (9). Further analyses with a large series may elucidate the nature of “osteogenic differentiation” in DDLPS.

### 2.2 Role of MRI in the diagnosis of DDLPS

**Figure 2:**
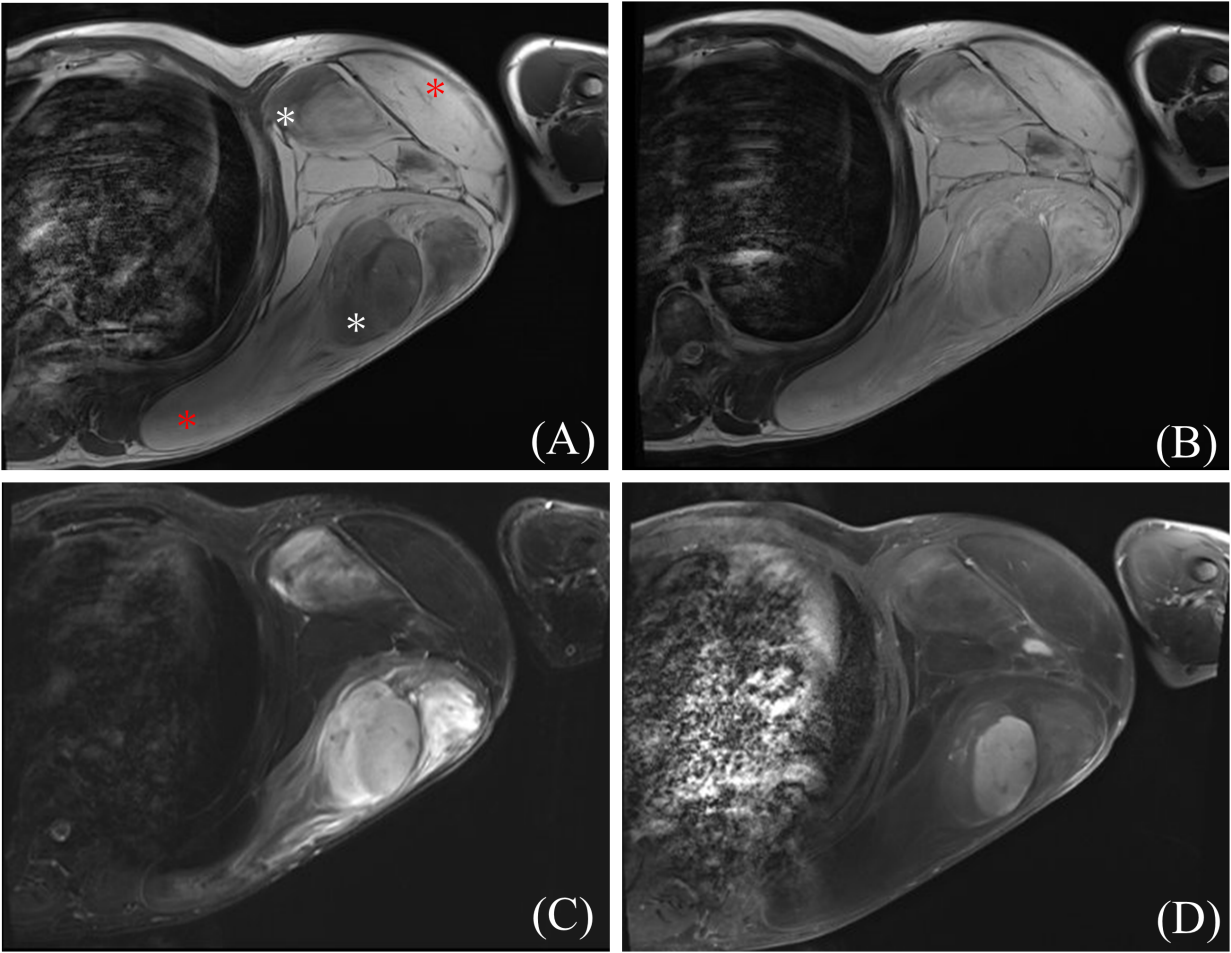
Representative magnetic resonance images of dedifferentiated liposarcoma (DDLPS). **(A)** T1-weighted image (white asterisk indicates DDLPS and red asterisk indicates well-differentiated liposarcoma components), **(B)** T2-weighted image, **(C)** short tau inversion recovery image, and **(D)** gadolinium-enhanced T1-weighted image.

MRI is a novel diagnostic modality for adipose tumors, regardless of whether they are benign or malignant. DDLPS often contains an ALT/WDLPS component, and the detection of the ALT/WDLPS component by MRI contributes largely to the differential diagnosis (**Figure 2**) (2). Owing to the abundance of fat tissue in the retroperitoneum, the distinction between adipose tissue or lipoma and ALT/WDL, even by MRI alone, is often difficult; however, the combination of MRI and other factors, including diameter, the presence or absence of septa, and contrast effects, increases both the sensitivity and specificity for diagnosing ALT/WDLPS (10). In fact, in this case, fat tissue was present at the edge of the tumor or surrounding the tumor on computed tomography (CT). Considering that these fat tissues may be part of the tumor, the ALT/WDL component, MRI can be used to determine the extent of surgical resection. However, caution is required, as the degree of fat content on MRI varies in each case of DDLPS; it was reported that 24% of cases had a high fat content, while 44% of cases had no fat content at all (2). Based on these findings, it would be extremely difficult to diagnose DDLPS without a fat component using MRI alone, even if it is of retroperitoneal origin. On the other hand, undoubtedly, CT is beneficial for detecting ossification and calcification. In cases of ossification without a connection to the skeletal system, extraskeletal osteosarcoma (EO) should always be considered a differential diagnosis (11). Imaging features of EO, including various degrees of necrosis, hemorrhagic changes on MRI in almost all cases, and calcification on CT in approximately 60% of cases, have been reported (12). Thus, MRI in DDLPS is beneficial for differentiating EO.

### 2.3 Role of histopathology in the diagnosis of DDLPS

**Figure 3:**
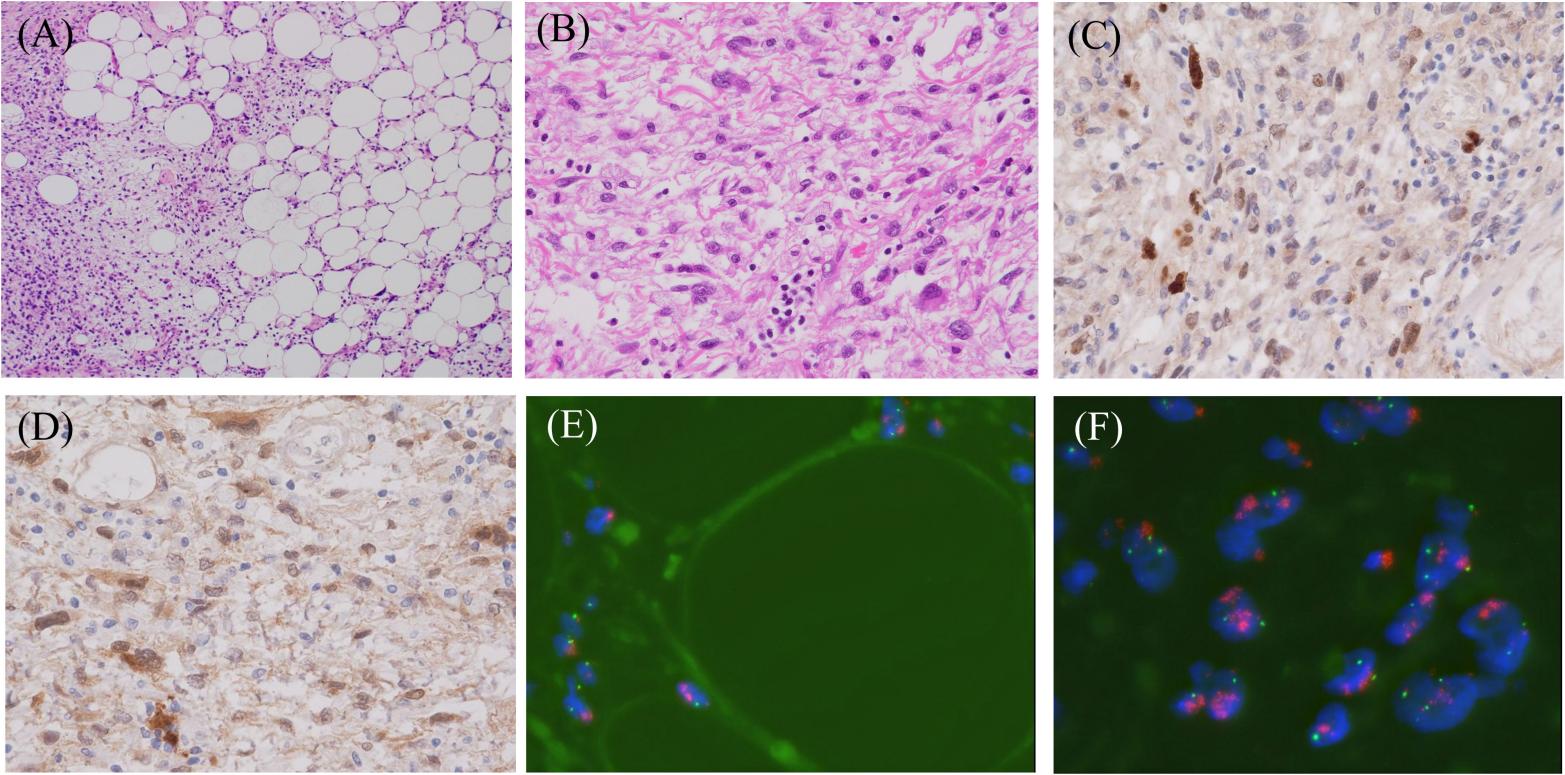
Representative histopathological and fluorescence *in situ* hybridization (FISH) images of dedifferentiated liposarcoma. **(A)** Hematoxylin and eosin staining shows the admixture of well-differentiated and dedifferentiated liposarcoma elements (magnification ×100), **(B)** The higher magnification (×400) of dedifferentiated liposarcoma composed of spindle and/or pleomorphic cells. Immuno-positivities for MDM2 **(C)** and CDK4 **(D)** in this lesion (magnification ×400). FISH analyses demonstrate MDM2 amplification in both well-differentiated **(E)** and dedifferentiated **(F)** areas. Red: MDM2 locus at 12q15, Green: centromere of chromosome 12 (SE 12/D12Z3). (objective lens magnification ×64).

The genetic features of DDLPS and ALT/WDLPS overlap; both share high-level amplification in the chromosomal 12q13-15 region, resulting in consistent amplification of *MDM2* and *CDK4* (3). Considering these features, IHC for MDM2 and CDK4 and FISH for MDM2 have great advantages for the diagnosis of both DDLPS and ALT/WDLPS, with a sensitivity of 100% for MDM2 and 83% for CDK4 in IHC (**Figure 3**) (13). On the contrary, high MDM2 and low CDK4 were observed in some cases, suggesting that the expression level of MDM2 does not coincide with the level of CDK4 (14). Regarding MDM2, the specificity may be high in cases with differentiating myxoid liposarcoma and pleomorphic liposarcoma because these liposarcomas almost always show negativity (15), although they may be low in cases with differentiating myxofibrosarcoma and undifferentiated pleomorphic sarcoma because these sarcomas occasionally show positivity (13). Therefore, combining MDM2 and CDK4 with p16 contributes to an accurate diagnosis because of its higher specificity (13, 15). However, we must be very careful in its interpretation because p16 may be positive, even in benign lipomas (13). FISH is more beneficial than IHC in diagnosing DDLPS and ALT/WDL, with reported positivity rates of 93% and 95%, respectively (16). The correlation between *MDM2* gene status and prognosis remains controversial because high *MDM2* amplification correlates with a shorter OS, while *MDM2* copy number (MDM2 to CEP12 ratio) does not correlate with metastasis and/or disease-related death (17, 18). Taken together, although further studies are needed, MDM2 may serve as a prognostic factor. Interestingly, a case of FISH positivity and IHC negativity for MDM2 has been reported, suggesting that when ALT/WDL or DDLPS is clinically suspected, not only IHC but also FISH should be performed (19). In cases with calcification, differentiation from EO is crucial not only in imaging but also in histopathology. Even with a pathological diagnosis, it is difficult to differentiate DDLPS from EO because the positivity rates of MDM2 and CDK4 in IHC and MDM2 in FISH for EO were reported to be 37%, 47%, and 38%, respectively, resulting in overlap between DDLPS and EO (20). On the other hand, the significance of *MDM2* amplification in EO remains controversial because of its occurrence in low-grade EO and low-grade EO with high-grade transformation, as well as the lack of association in some low-grade EO cases. Larger studies are needed to clarify the role of 12q13-15 amplification, including *MDM2*, in EO (21). Although SATB2, an osteoblast marker, has been reported to be useful in EO (11), its usefulness and specificity in differentiating EO from DDLPS with osteosarcomatous elements remain unclear. In cases of DDLPS with no ALT/WDL component but with ossification, the diagnosis should be based on the overall histopathological findings with IHC and FISH, with attention to differentiation from EO.

### 2.4 Treatment strategy

The principal treatment is surgery, with R0 or R1 surgery preferred if possible; R0 and R1 surgeries are associated with lower recurrence and a better prognosis than R2 surgery (22). In the retroperitoneum, important organs, such as the kidneys and intestinal tract, often make it difficult to complete R0 surgery. Therefore, adjuvant therapies, including chemotherapy and radiation therapy, are often used. Preoperative radiotherapy contributed to better local control in grades 1 and 2 DDLPS but did not affect OS. Furthermore, preoperative radiotherapy did not affect local control or OS in patients with grade 3 DDLPS (23). Chemotherapy with doxorubicin, either as a single agent or in combination with ifosfamide, has been reported. Although the combination of doxorubicin and ifosfamide was expected to have clinical effects, there was no significant improvement in the OS; however, side effects, such as hematological toxicity, were frequently observed (4). Recently, clinical trials of agents targeting MDM2 and CDK4, which are characteristic of ALT/WDL and DDLPS, have been conducted (24). Vanni et al. identified CDK4 as a prognostic biomarker and reported the synergistic effect of CDK4 inhibitors with lenvatinib (14). Besides agents targeting MDM2 and CDK4, tyrosine kinase receptor inhibitors, peroxisome proliferator-activated receptor gamma agonists, and nelfinavir were considered candidates (24). If the efficacy of these novel agents is confirmed through further clinical trials, particularly when combined with radiation and chemotherapy, they could be applied to reduction surgery and inoperable cases.

## 3 Conclusion

Herein, we introduce recent diagnostic and therapeutic advances in DDLPS. Although typical cases of DDLPS with a retroperitoneal origin and an ALT/WDLPS component can be diagnosed using imaging, the necessity for a pathological diagnosis remains largely unchanged. Furthermore, in cases of DDLPS with heterogeneous changes, such as myogenic osteosarcomatous elements, caution should be exercised in the differential diagnosis. The role of MDM2 in IHC and FISH is crucial not only in diagnosis but also in therapeutic applications.
